# *Sesn-1* is required for lifespan extension during caloric deprivation in *C. elegans* through inhibition of mTORC1 and activation of autophagy

**DOI:** 10.18632/aging.206290

**Published:** 2025-07-28

**Authors:** Andrei O. Zheltukhin, Peter M. Chumakov, Andrei V. Budanov

**Affiliations:** 1 Engelhardt Institute of Molecular Biology, Russian Academy of Sciences, Moscow, Russia; 2 School of Biochemistry and Immunology, Trinity Biomedical Sciences Institute, Trinity College Dublin, Dublin 2, Ireland; 3 Shemyakin-Ovchinnikov Institute of Bioorganic Chemistry of the Russian Academy of Sciences, Moscow, Russia

**Keywords:** *sesn-1*, mTOR, autophagy, aging

## Abstract

Sestrins, evolutionarily conserved stress-responsive proteins, are increasingly recognized for their potential role in lifespan regulation. This study aimed to elucidate the influence of the *sesn-1* gene on lifespan modulation during caloric deprivation (CD) in the model organism *C. elegans*. Our findings reveal that *sesn-1* mediates lifespan extension under CD, primarily through the repression of mTORC1 kinase and activation of autophagy. Moreover, we identified an essential role for *sesn-1* in enhancing stress resilience in nematodes, particularly in the context of nutrient sensing. Further investigations demonstrated *sesn-1*’s interaction with the GATOR2 protein complex, its role in maintaining muscle integrity and a potential synergy between *sesn-1* and the FOXO pathway. Overall, our research underscores the profound implications of Sestrins in aging and stress resistance, shedding light on possible therapeutic avenues for prevention and treatment of age-associated disorders.

## INTRODUCTION

Sestrins were identified two decades ago as stress-responsive proteins that play an important role in regulating cellular homeostasis. Vertebrate genomes showcase three Sestrin genes (*SESN1-3*), while invertebrates feature just one [1–3]. Numerous stressors, ranging from hypoxia and oxidative stress to DNA damage and nutrient deprivation, induce Sestrin expression in mammalian cells. The orchestration behind this expression involves several transcription factors, notably p53, FOXO, ATF4 and NRF2 [4–6]. Highlighting evolutionary conservation [7], the same signalling pathways trigger the activation of *dSesn* in *D. melanogaster* [8]. Consequently, Sestrins play pivotal roles in the regulation of cellular viability under various stress conditions, such as hypoxia, oxidative stress, DNA damage and glucose deprivation [2, 9–13].

Earlier research from our team established Sestrins as antioxidant proteins that play a critical role in inhibiting the mechanistic target of rapamycin complex 1 (mTORC1) kinase [9, 14–16]. mTORC1 is an intricate environmental sensor that integrates signals from nutrients, growth factors and stresses to regulate cell fate decisions. mTORC1 functions as a central regulator of biosynthesis and cell growth, while also suppressing macroautophagy (herein in the text – autophagy) [17]. Autophagy is the process of encapsulating intracellular components into autophagosomal vesicles, followed by the degradation of their contents in lysosomes. Autophagy is essential for nutrient supply and cell repair under stressful conditions. While autophagy typically supports cell survival under stress, it can also trigger autophagy-dependent cell death [18].

Remarkably, mTORC1 plays a key role in lifespan and aging regulation across various species. Application of specific mTORC1 inhibitors, like rapamycin, has been shown to enhance lifespan in different organisms from yeast to mice [19–23]. Similarly, caloric restriction (CR), a well-documented longevity enhancer across many species, also represses mTORC1 activity, further cementing the role of this kinase in aging control [24–26]. Nutrient and energy availability signals are transmitted to mTORC1 through the evolutionarily conserved insulin/IGF1 signaling pathway in metazoans [27]. In addition to mTORC1, the insulin/*IGF1* signalling pathway inhibits the forkhead box O (*FoxO*) transcription factors via Akt-mediated phosphorylation and nuclear exclusion [28], and FoxOs regulate adaptation to starvation conditions in metazoans [29, 30]. In contrast, mTORC1 is activated by Akt in response to insulin/IGF-1 signaling and promotes anabolic processes while inhibiting catabolic pathways [31].

Branched-chain amino acids (BCAA), especially leucine, play a critical role in the regulation of mTORC1, primarily via activation of the GATOR2 protein complex. GATOR2 is an inhibitor of the GATOR1 protein complex, which suppresses the activity of RagA/B GTPases, thereby preventing mTORC1 translocation to lysosomes and its activation [17]. Sestrins inhibit mTORC1 through the interaction with GATOR2. Leucine binding to Sestrins induces conformational changes that weaken the interaction between Sestrins and GATOR2, leading to mTORC1 activation [32, 33].

Being regulators of stress response and metabolism, broader implications of Sestrins in aging control cannot be understated. In *C. elegans*, variations in *sesn-1* expression levels have measurable effects on lifespan and physiological functions [34, 35]. Similarly, in *D. melanogaster*, alterations in dSesn levels contribute to the development of age-associated disorders such as muscle degeneration and cardiac arrhythmia [8]. Many of Sestrin’s effects might be attributed to its role in activating autophagy through the mTORC1-mediated mechanism. Recent findings indicate that dSesn plays a key role in extending lifespan in flies subjected to BCAA restriction [36]. Stem cell functionality is maintained through autophagy [37] and the impact of Sestrins on various facets of stem cell biology, encompassing both stemness and differentiation, is also being recognized [38].

In an effort to elucidate overarching role of Sestrins in lifespan modulation during caloric deprivation (CD), we utilized a *C. elegans* model in which *sesn-1* was inactivated via gene deletion [35]. Existing research has underscored the remarkable lifespan extension in nematodes upon CD [39, 40]. Our studies aimed to elucidate Sestrin’s role in lifespan regulation under CD and to examine how *sesn-1* deficient worms respond to stress. Based on our studies in *C. elegans*, we demonstrate that the relationship between *sesn-1* and the GATOR–TORC1–autophagy axis is highly conserved across eukaryotes [7]. We investigated the involvement of *sesn-1* in signalling pathways that link mTORC1 activity, autophagy and increased lifespan during CD in *C. elegans*, such as those regulated by *let-363* (an ortholog of the mammalian m*TOR gene*) [41], *daf-2* (an ortholog of the mammalian *IGFR1* gene) [42] and *daf-16* (an ortholog of the mammalian *FOXO* genes) [42]. We also evaluated the potential involvement of *sesn-1* in lifespan extension in nematodes carrying a deletion in the *eat-2* gene [43] that is required for proper pharyngeal function. Animals carrying this mutation experience continuous, moderate caloric restriction throughout development and adulthood. Our findings confirm the critical role of *sesn-1* in lifespan extension mediated by mTORC1 inhibition and autophagy activation in response to CD.

## RESULTS

### *Sesn-1* modulates lifespan extension in *C. elegans*

**Figure 1:**
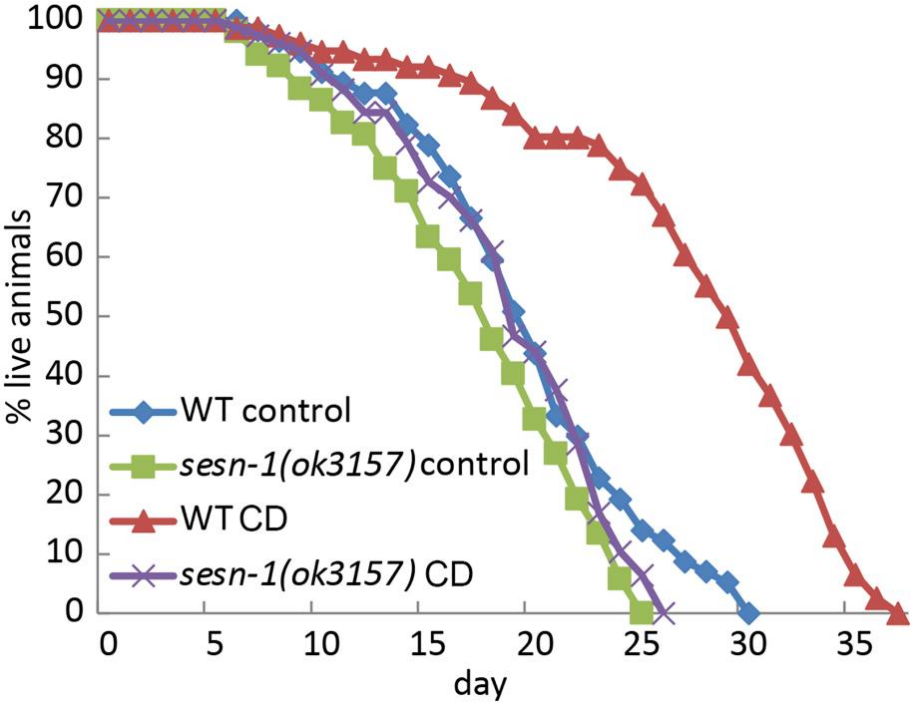
***C. elegans* lifespan extension under CD is modulated by *sesn-1*.** The lifespan of WT and *sesn-1(ok3157)* nematodes was assessed after plating on control or axenic media.

To understand the role of *sesn-1*, the nematode ortholog of the Sestrin genes, in the control of aging and lifespan, we determined its impact on lifespan regulation under CD conditions. Using established protocols [40], nematodes were cultured on agar plates with (control) or without bacteria to assess lifespan. As previously reported [34], *sesn1*-deletion mutant strain, *sesn-1(ok3157),* exhibited a marginally reduced lifespan compared to their wild-type (WT) counterparts under normal conditions. This suggests that *sesn-1* facilitates nematode homeostasis in food-abundant conditions. However, in our observations, the lifespan of the *sesn-1(ok3157)* animals did not differ from that of the WT counterparts under *ad libitum* conditions (Figure 1 and Table 1). Therefore, we decided to examine the role of *sesn-1* in the regulation of lifespan in response to CD. According to our data, CD augmented the lifespan of WT animals by 40.2%. In contrast, the *sesn-1(ok3157)* worms experienced a mere 6.2% increase in lifespan, underscoring the pivotal role of *sesn-1* in lifespan extension following CD exposure (Figure 1 and Table 1). The same data were observed in another *sesn-1* deficient strain, *sesn-1(ie24589)* (IE24589 strain with MOS-1 transposon insertion in 3 exon) (Supplementary Figure 1).

**Table 1:** Lifespan extension means analysis for control WT and *sesn-1(ok3157)* nematodes under caloric deprivation, with RNAi expression against *npp-18* and *Y32H12A.8*.

**Strain**	**RNAi**	**Control mean lifespan ± SEM, days**	***n***	**Starvation mean lifespan ± SEM, days**	***n***	**Effect vs. control %**	***p*-value vs. control**
WT	EV	18,1 ± 0,50	46	24,5 ± 0,61	57	+ 40,2%	<0,0001
*sesn-1(ok3157)*	EV	17,6 ± 0,52	49	18,8 ± 0,6	57	+ 6,19%	0,8242
WT	*npp-18*	17,5 ± 0,47	52	18,3 ± 0,58	49	+ 4,38%	>0,9999
*sesn-1(ok3157)*	*npp-18*	16,7 ± 0,44	51	17,3 ± 0,54	52	+ 3,95%	>0,9999
WT	*Y32H12A.8*	17,1 ± 0,59	50	17,8 ± 0,71	46	+ 3,5%	>0,9999
*sesn-1(ok3157)*	*Y32H12A.8*	16,5 ± 0,53	50	16,5 ± 0,45	44	+ 0,12%	>0,9999

### *Sesn-1* protects nematodes from multiple stresses

**Figure 2:**
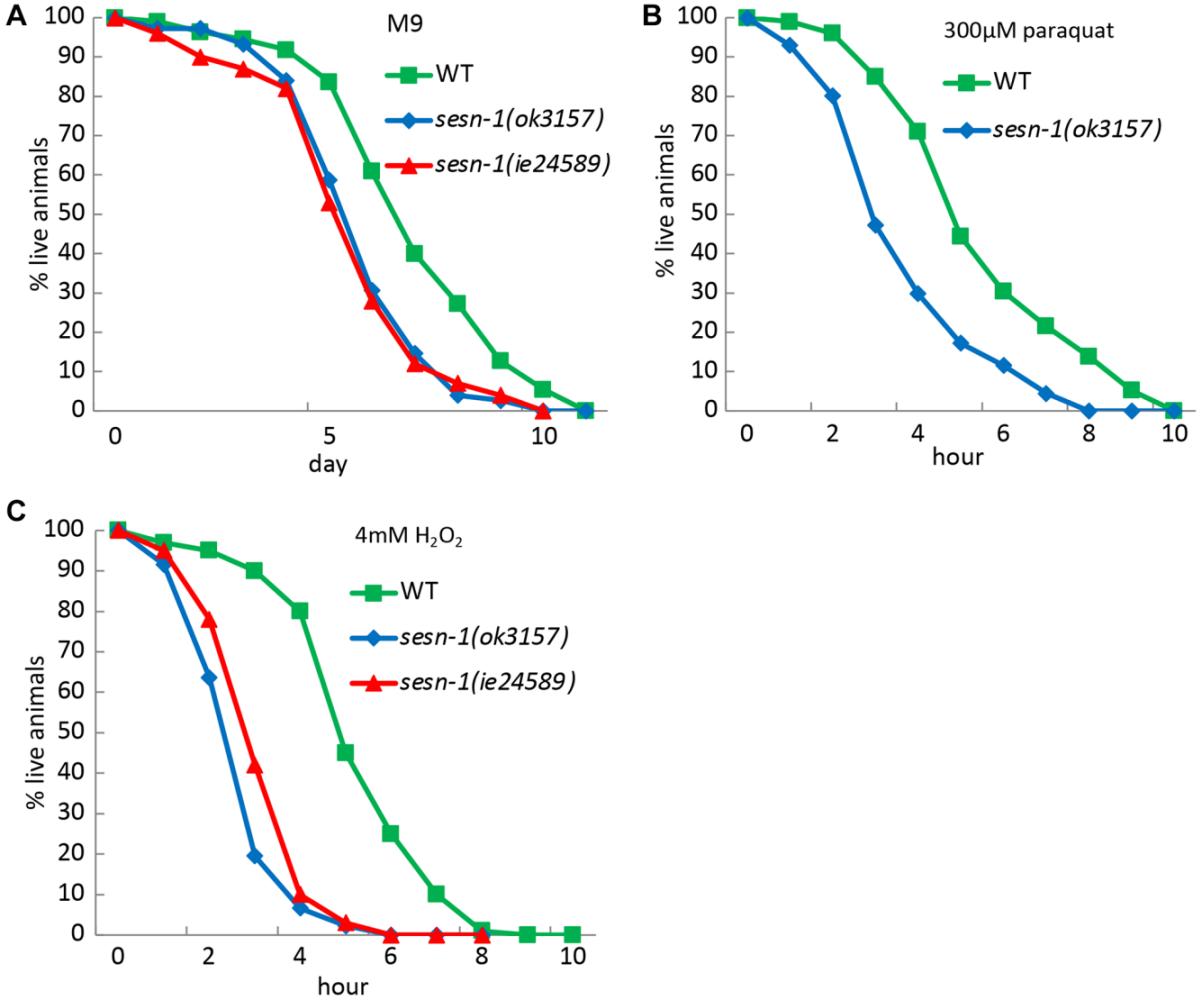
**Role of *Sesn-1* in stress resistance.** The viability of WT and *sesn-1(ok3157) C. elegans* was studied under various stress conditions. (**A**) In axenic M9 medium (*n* = 783 for WT, *n* = 437 for *sesn-1(ok3157), n* = 511 for *sesn-1(ie24589)*, the mean survival rates for *sesn-1(ok3157)* and *sesn-1(ie24589)* were 6.13 ± 0.4 and 6.16 ± 0.3 days, respectively, compared to 7.9 ± 0.5 days for WT. The difference between *sesn-1(ok3157)* and *sesn-1(ie24589)* was not significant (*P* > 0.05), while both mutants showed significantly lower survival than WT (*P* < 0.001). (**B**) In the presence of 300 μM paraquat (*n* = 1103 for WT, *n* = 876 for *sesn-1(ok3157)*, *n* = 882 for *sesn-1(ie24589)*), the mean survival rates were 4.1 ± 1.2 and 4.0 ± 0.9 hours for *sesn-1(ok3157)* and *sesn-1(ie24589)*, respectively, compared to 5.8 ± 1.2 hours for WT. Again, the difference between the two *sesn-1* mutants was not significant (*P* > 0.05), while both were significantly more sensitive than WT (*P* < 0.001). (**C**) In the presence of 4 mM H2O2 (*n* = 1301 for WT, *n* = 930 for *sesn-1(ok3157)*, *n* = 827 for *sesn-1(ie24589)*), the mean survival rates were 3.3 ± 0.5 and 3.8 ± 0.6 hours for *sesn-1(ok3157)* and *sesn-1(ie24589)*, respectively, compared to 6.4 ± 0.8 hours for WT. The difference between *sesn-1(ok3157)* and *sesn-1(ie24589)* was not statistically significant (*P* > 0.05), while both mutants showed significantly reduced survival compared to WT (*P* < 0.001). Data are presented as mean ± S.E.M.

Given the link between stress response and lifespan modulation, where proteins responsive to stress curb accumulation of age-linked damage, we investigated how *sesn-1* influences stress tolerance. Exposing nematodes to various stressors like oxidizing agents (paraquat and hydrogen peroxide) and axenic culture medium (M9) revealed stark differences between WT and *sesn-1(ok3157)* or *sesn-1(ie24589)* animals. When WT first larval stage (L1) nematodes were placed in M9 medium, they outlived their *sesn-1*(ok3157) or *sesn-1*(ie24589) congeners, showing reduced resistance of *sesn-1* mutants to nutrient restriction (Figure 2A). Furthermore, oxidative stress induced by paraquat and H_2_O_2_ dramatically accelerated the death of *sesn-1(ok3157)* and *sesn-1(ie24589)* worms compared to their WT counterparts (Figure 2B, 2C). These collective findings emphasize the role of *sesn-1* in bolstering stress resilience in nematodes, likely through mechanisms analogous to those that promote lifespan extension during CD.

### *Sesn-1*: an essential component for autophagy activation

**Figure 3:**
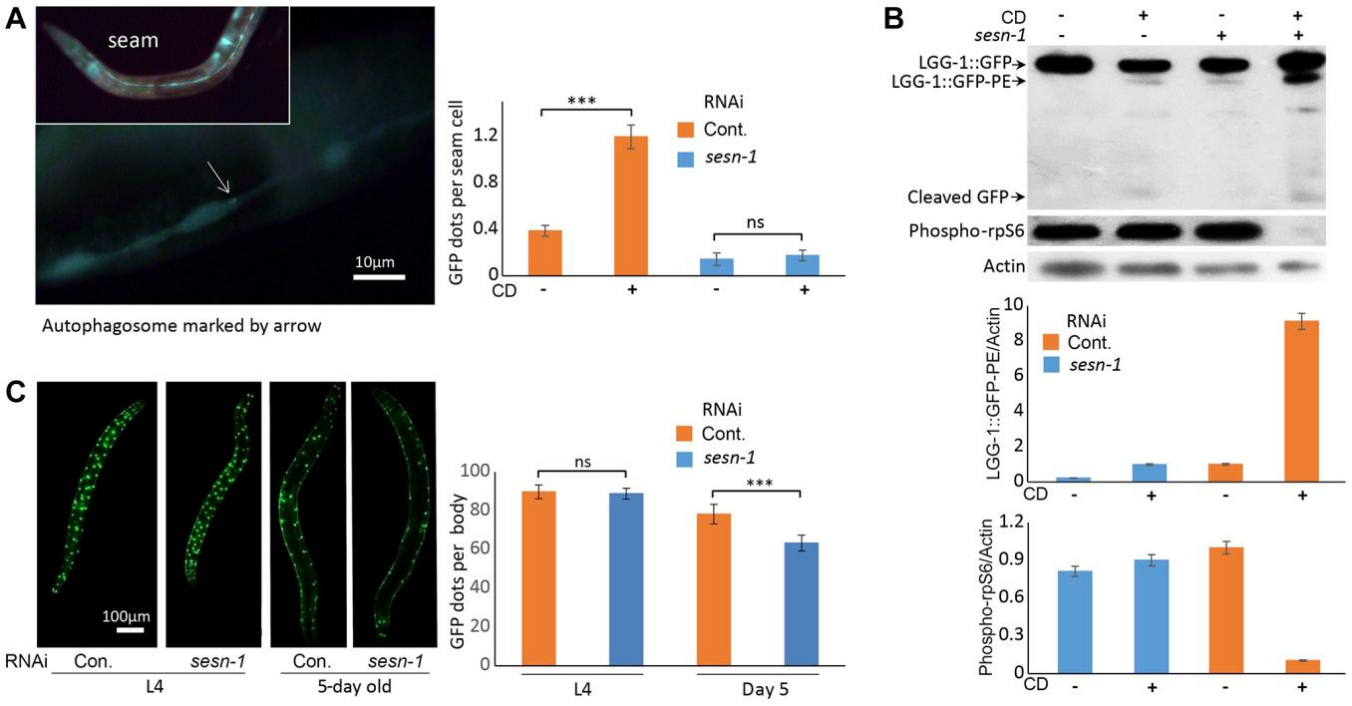
**Autophagy activation by *sesn-1* under CD correlates with reduced muscle degeneration.** (**A**) Autophagosome accumulation in seam cells. Both control *adIs2122* (DA2123 strain) and *adIs2122; sesn-1(RNAi)* nematodes, expressing a GFP-tagged LGG-1 fusion protein during L3, were exposed to axenic medium. Autophagosome counts per seam cell were analyzed under control conditions (*n* = 137 for *adIs2122*, *n* = 56 for *adIs2122; sesn-1(RNAi)*) and starvation conditions (*n* = 117 for *adIs2122*, *n* = 80 for *adIs2122; sesn-1(RNAi)*). “ns” and “^***^” indicate *P*-values > 0.05 and < 0.001, respectively. All bar graphs are presented as mean ± S.E.M. (**B**) Immunoblot and densitometric analyses showing relative levels of GFP::LGG-1, its phosphatidylethanolamine-conjugated form (LGG-1::GFP-PE), and the phosphorylated form of ribosomal protein S6 (phospho-rpS6) in *adIs2122* (DA2123 strain) and *adIs2122; sesn-1(RNAi)* worms. All bar graphs represent blot intensity normalized to actin. (**C**) Whole-body images of nematodes expressing a *myo-3p::GFP* NLS-tagged fusion protein in body wall muscle nuclei in the *ccIs4251* (PD4251 strain). Nuclear counts were performed at L4 (*n* = 24 for *ccIs4251* and *n* = 23 for *ccIs4251; sesn-1(RNAi)*) and at 5 days of adulthood (*n* = 20 for both groups). “ns” and “^***^” indicate *P*-values > 0.05 and < 0.001, respectively. All bar graphs are presented as mean ± S.E.M.

Autophagy, a mechanism that promotes lifespan extension during nutrient scarcity and augments stress resistance [44, 45], may be activated by *sesn-1*. To test this hypothesis, we utilized a C. elegans strain *adls2122* that expresses a GFP-tagged LGG-1 fusion protein (LGG-1::GFP), where LGG-1 is the nematode ortholog of the mammalian autophagy marker LC3. Autophagosomes incorporating LGG-1::GFP form discrete GFP-positive vesicles, which can be readily visualized by fluorescence microscopy. [46]. In WT third larval stage (L3) nematodes subjected to CD, we observed a pronounced accumulation of LGG-1::GFP-labeled autophagosomes within the seam cells. In contrast, nematodes with *sesn-1* silenced by RNAi (*sesn-1(RNAi)*) exhibited only a modest increase in LGG-1::GFP-labeled autophagosomes, emphasizing *sesn-1*’s crucial role in autophagy initiation during starvation. Under control conditions, WT animals exhibited an average of 0.38 autophagosomes per cell compared to just 0.14 autophagosomes per cell in *sesn-1* mutants—a difference of more than two-fold, which was statistically significant (*p* = 0.02194) (Figure 3A). In mammals, Sestrin-dependent autophagy activation is mediated by mTORC1 inhibition [6]. To ascertain *sesn-1*’s role in autophagy during nematode starvation, we subjected both WT and *sesn-1(RNAi)* worms to CD and evaluated mTORC1 activity and autophagy levels using immunoblotting. While the control group exhibited reduced ribosomal protein S6 phosphorylation post-CD exposure — an mTORC1-inhibiting event — this reduction was not observed in *sesn-1(RNAi)* animals (Figure 3B). Evaluation of autophagy by comparing LGG-1::GFP with its pro-autophagic, phosphatidylethanolamine-conjugated form (LGG-1::GFP-PE) revealed that CD prompted substantial accumulation of LGG-1::GFP-PE in WT worms. Yet, *sesn-1(RNAi)* worms exhibited only a minor increase in this autophagosome marker post-starvation, again highlighting *sesn-1*’s indispensable role in autophagy modulation under CD in *C. elegans* (Figure 3B). In a bid to elucidate *sesn-1* contribution to autophagosome formation, we evaluated LGG-1::GFP-PE formation intensity in worms pre-treated with 200 mM chloroquine for 24 hours [47, 48]. Under acute starvation, WT worms manifested pronounced LGG-1::GFP-PE accumulation, but this was notably suppressed in *sesn-1(RNAi)* worms, signifying *sesn-1*’s necessity for appropriate autophagy activation (Supplementary Figure 2).

### Role of *Sesn-1* role in sustaining muscle integrity

Previous studies in *D. melanogaster* and mice have linked Sestrins to preservation of muscle function, primarily through their role in mitigating oxidative stress-induced damage [8, 38, 49]. To investigate *sesn-1*’s potential role in preserving muscle density in *C. elegans*, we utilized a nematode strain *(ccIs4251 I, e1282 IV)* expressing *myo-3p*::GFP NLS-tagged fusion protein, which labels myocyte nuclei. Assessment of muscle density across various developmental stages, particularly the fourth larval stage (L4) and 5-day-old adult stage, in WT and *sesn-1(RNAi)* animals revealed that muscle density was consistent in the L4 animals across the groups. However, a pronounced reduction in myocyte count was observed in 5-day-old *sesn-1(RNAi)* adult worms, highlighting the essential role of *sesn-1* in maintaining adult muscle function (Figure 3C).

### *Sesn-1* facilitates lifespan extension through GATOR2

**Figure 4:**
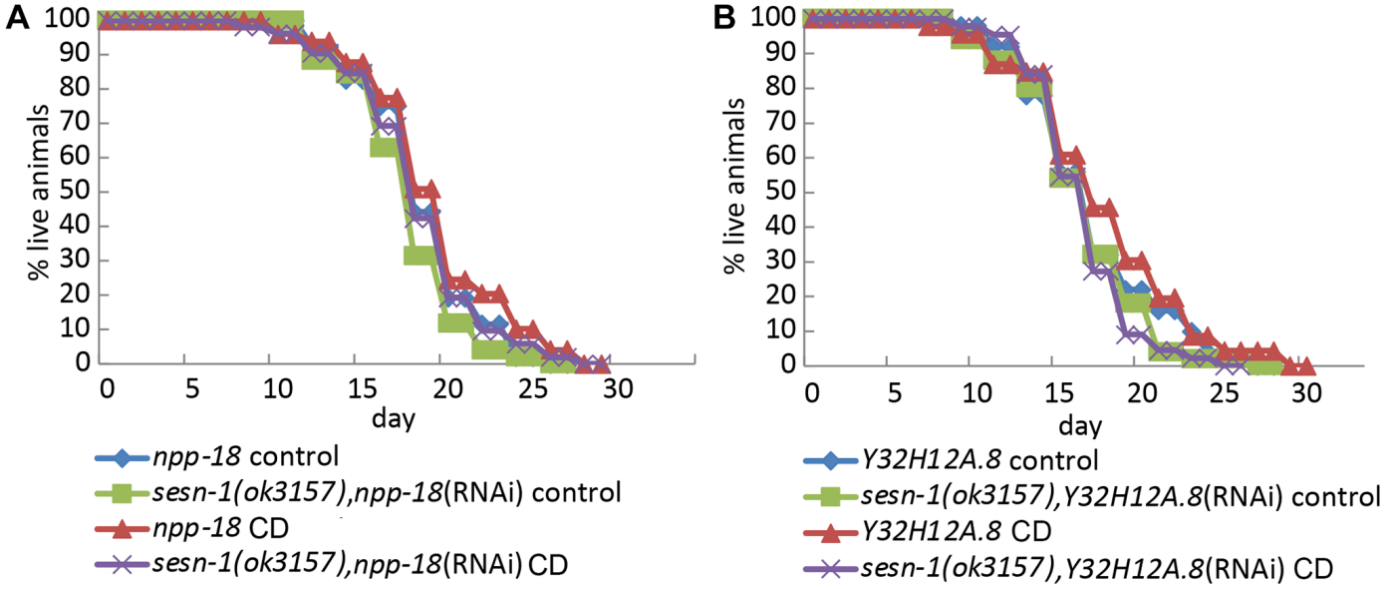
***Sesn-1* modulates longevity under CD via GATOR2.** (**A**) Lifespan of WT and *sesn-1(ok3157)* worms subjected to *npp-18* RNAi knockdown in *ad libitum* and axenic media. (**B**) Lifespan of WT and *sesn-1(ok3157)* nematodes subjected to *Y32H12A.8* RNAi knockdown in *ad libitum* and axenic media.

**Figure 5:**
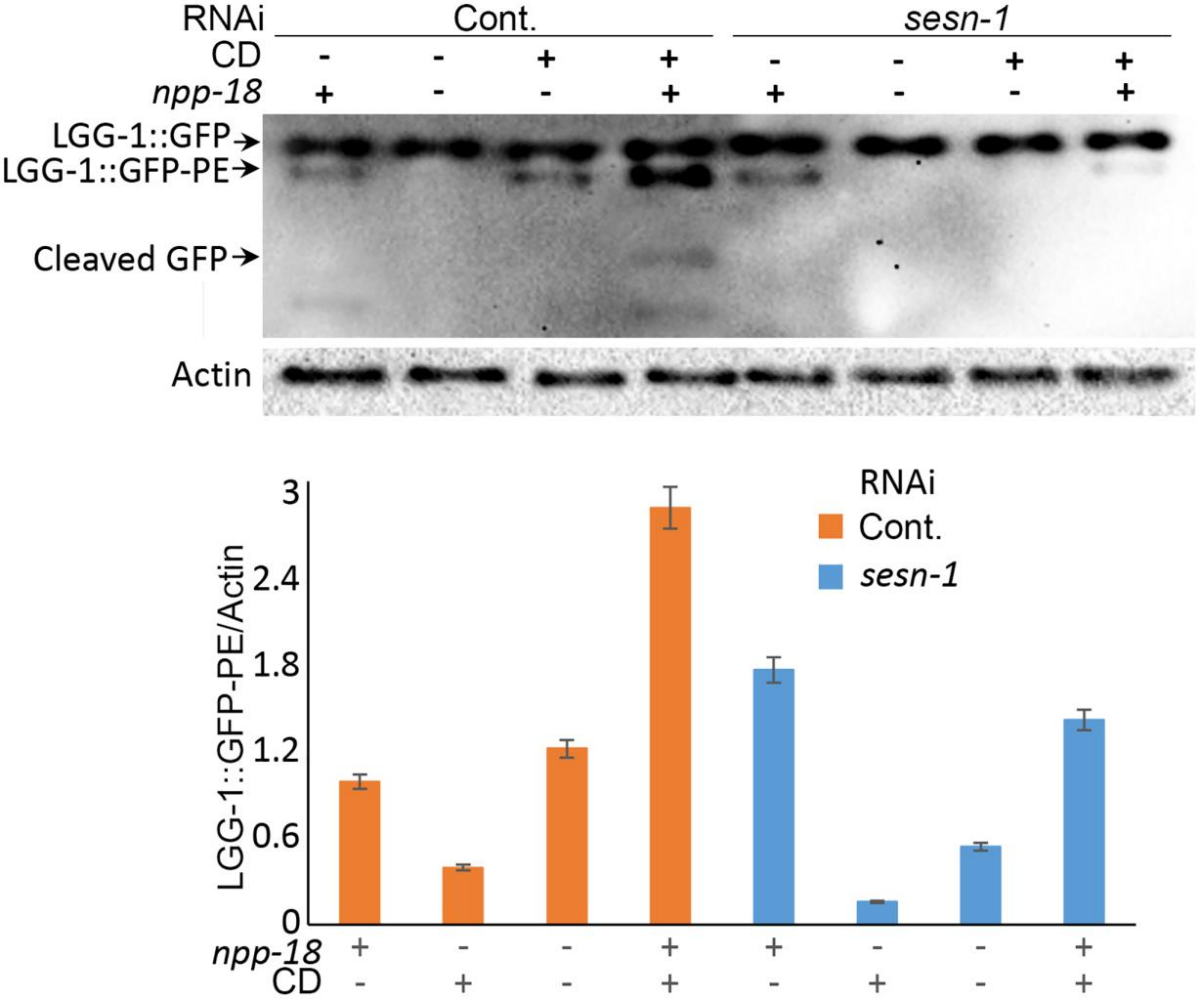
**Autophagy intensity in *C. elegans* is influenced by *sesn-1* and *npp-18*, a component of the GATOR2 complex.** Control *adIs2122* and *adIs2122; sesn-1(RNAi)* nematodes expressing GFP::LGG-1, with or without simultaneous *npp-18* knockdown by RNAi, were subjected to starvation. The relative levels of GFP::LGG-1 conjugation to autophagosomes were measured by immunoblot densitometry. All bar graphs represent blot intensity normalized to actin.

Previous studies in mammalian cells have shown that Sestrins suppress mTORC1 activity by inhibiting GATOR2 [32, 50]. The Sestrin-GATOR-mTORC1 signalling pathway is known to be conserved in eukaryotes [7], so we analysed the potential involvement of GATOR2 in the *sesn-1*-modulated lifespan extension. We proposed that if *sesn-1*’s effects on the lifespan extension are GATOR2-dependent, *sesn-1* would not significantly influence lifespan in GATOR2-deficient worms under CD. To test this, we used RNAi to silence the genes encoding the major components of GATOR2: *npp-18* and *Y32H12A.8*, the orthologs of the mammalian *SEH1L* and *WDR24* genes, respectively, in WT and *sesn-1(ok3157)* nematodes (Figure 4A, 4B) and measured lifespan increase during CD. While *sesn-1* facilitated lifespan extension during CD in control worms, its effects were notably diminished when *npp-18* and *Y32H12A.8* were suppressed (Figure 4A, 4B and Table 1). Further studies examining *sesn-1*’s role in autophagy regulation in worms with suppressed GATOR2, using immunoblotting, revealed intriguing findings. In the absence of *sesn-1*, worms still exhibited modest autophagy; however, simultaneous RNAi-mediated suppression of *sesn-1* and *npp-18* significantly reduced autophagy. This suggests that *sesn-1* likely operates via *npp-18* (Figure 5).

### *Sesn-1* supports lifespan extension via FOXO and in eat-2 mutants

**Figure 6:**
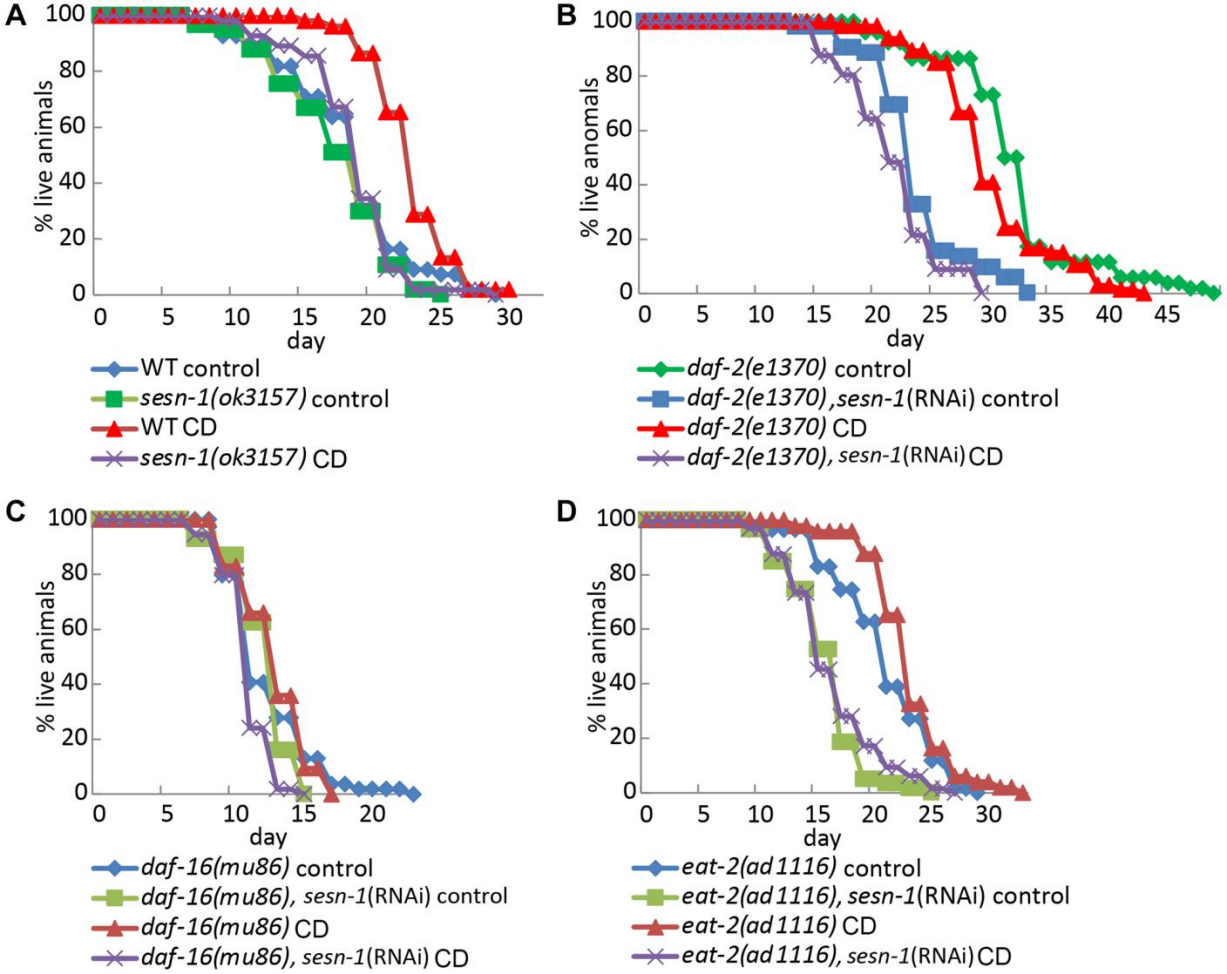
**Lifespan regulation by *sesn-1* through the *daf-16* pathway and in *eat-2* mutant under starvation.** Analysis of the lifespan of different *C. elegans* strains: (**A**) WT and *sesn-1(ok3157)*, (**B**) *daf-2(e1370)*, (**C**) *daf-16(mu86)* and (**D**) *eat-2(ad1116)*, with and without *sesn-1(RNAi)*, incubated under control or caloric deprivation (CD) conditions.

*Sesn-1* may be involved in the signaling pathways known to be controlled by CD in *C. elegans,* such as those regulated by *let-363*(*TOR*) [41, 51], *daf-2*(*IGF1R*) [42] and *daf-16*(*FOXO*) [42]. We also tested the possible involvement of *sesn-1* in lifespan extension in nematodes with a deletion in the *eat-2* (*ad1116*) gene [43]. We used nematode strains with knockouts of *eat-2*(*ad1116*), *daf-2*(*e1370*) and *daf-16*(*mu86*) either with or without *sesn-1* silencing by RNAi. The contribution of *sesn-1* to the FOXO pathway and lifespan extension in *eat-2(ad1116)* nematodes was appreciable but did not reach statistical significance (Figure 6A–6D and Table 2). Without *daf-16* and *sesn-1*, the lifespan of starved worms is reduced by 9.5% (Figure 6C), whereas the presence of *sesn-1* increases lifespan of *daf-16*(*mu86*) worms by 7.5% under CD conditions. Survival of the *eat-2* worms under CD requires sufficient autophagic activity. In starved nematodes lacking both *eat-2* and *sesn-1*, lifespan increased by 3.6%, whereas the presence of *sesn-1* extended the lifespan of *eat-2(ad1116)* mutants by 11% (Figure 6D and Table 2).

**Table 2:** Lifespan extension means analysis under CD for *daf-2* (*e1370*), *eat-2* (*ad1116*), *daf-16* (*mu86*) nematodes with *sesn-1* RNAi expression and WT.

**Strain**	***Sesn-1* gene status**	**Ortholog**	**Control mean lifespan ± SEM, days**	***n***	**Starvation mean lifespan ± SEM, days**	***n***	**Effect vs. control, %**	***p*-value vs. control**
WT	WT		18,2 ± 0,64	55	22,8 ± 0,39	52	+25,3	<0,0001
*sesn-1(ok3157)*	deletion		17,3 ± 0,56	51	18,6 ± 0,48	55	+8,7	0,4054
*daf-2 (e1370)*	WT	IGF1r	31,8 ± 0,84	53	29,9 ± 0,62	66	−6,1	0,0252
*daf-2 (e1370)*	RNAi	IGF1r	23,4 ± 0,56	52	21,4 ± 0,52	56	−8,7	0,0219
*eat-2 (ad1116)*	WT		20,8 ± 0,58	59	23,1 ± 0,5	49	+11,0	0,0057
*eat-2 (ad1116)*	RNAi		15,8 ± 0,41	59	16,3 ± 0,46	64	+3,6	<0,0001
*daf-16 (mu86)*	WT	FOXO family	12,4 ± 0,41	54	13,3 ± 0,34	53	+7,5	>0,9999
*daf-16 (mu86)*	RNAi	FOXO family	12,2 ± 0,25	69	11,0 ± 0,22	55	−9,5	0,7069

## DISCUSSION

Aging is one of humanity’s most pressing challenges, exacerbated by the global rise in life expectancy and the parallel increase in age-related diseases such as cancer, diabetes and neurodegenerative disorders. Understanding the complexities of aging is crucial for mitigating its adverse effects on human well-being and ensuring healthy aging. Notably, the key signaling pathways that govern aging are evolutionary conserved. Model organisms like *C. elegans* serve as indispensable tools for unravelling the molecular mechanisms underlying aging. CR has emerged as a key physiological intervention that extends both lifespan and healthspan across a wide range of eukaryotic species. In nematodes, CR as well as CD mimic the lifespan-extending effects observed in worms with the *eat-2*(*ad1116*) mutation, which significantly restricts food intake [43]. Interestingly, overlaying CD and CR on *eat-2* mutants does not further enhance this lifespan extension (Figure 6D and Table 2), suggesting that the *eat-2* mutation, CD and CR may share common mechanisms of lifespan regulation.

The central role of mTORC1, a critical nutrient sensor that is deactivated by glucose and amino acid scarcity, is evident in the aging regulation across diverse species from yeast to mice [25]. Both CR and inhibition of mTORC1 similarly extend lifespan, implying a common underlying mechanism [24]. Autophagy activation is a critical mechanism of lifespan extension, as evidenced by the abatement of lifespan extension in animals with inhibited autophagy during CR [41]. In *C. elegans*, this lifespan enhancement coincides with increased stress tolerance, a benefit attributed to activated autophagy, particularly during food scarcity [52, 53]. Adequate activation of autophagy by Sestrins may underlie the phenomenon of hormesis, the adaptive response to severe stress following exposure to low-level stress, which could also promote lifespan and healthspan extension [53].

Various sensors activated by different nutrients and stress factors likely modulate autophagy, stress response and longevity. Essential proteins, including Sestrins, may integrate these signals and direct them to mTORC1-regulated autophagy, enabling an adaptive response to changing environments. In mammals, glucose shortage and amino acid deprivation increase *SESN2* expression via a mechanism mediated by ATF4 [4, 54]. Meanwhile, oxidative stress, DNA damage and hypoxia stimulate Sestrins’ expression through p53, NRF2 and some other transcription factors [37, 55, 56]. Interestingly, in our studies, *sesn-1* mRNA levels peaked at 10 hours post-CD initiation but declined by 16 hours, suggesting potential post-transcriptional regulation (data not shown). This could be explained by a decoupling of SESN-1 protein and mRNA expression, with the protein potentially remaining stable despite the reduction in mRNA levels – similar to the dynamics observed for the mammalian SESN2 protein after 24 hours of H2O2 exposure [9]. Such protein stabilization, coupled with its mRNA downregulation during continuous stress, may serve an adaptive function, conserving energy and optimizing recovery when conditions improve. Deletion of *sesn-1* significantly attenuated CD-induced lifespan extension, highlighting its critical role in this process (Figure 1) [35, 57]. The slight extension observed in *sesn-1*-null animals subjected to CD might arise from Sestrin-independent nutrient sensing effects of other unidentified pathways.

Given autophagy’s strong links to longevity and stress mitigation [44], we investigated *sesn-1*’s involvement in regulating autophagy and its key effector, mTORC1 [41, 51], in the context of CD. Our findings demonstrate that *sesn-1* mediates mTORC1 suppression and autophagy activation during food scarcity, potentially driving lifespan extension and stress resilience (Figures 1 and 2A–2C). Failure of *sesn-1 (RNAi)* worms to activate autophagy properly in response to starvation (Figure 3B) and activate autophagosome formation at the appropriate level in response to chloroquine exposure (Supplementary Figure 2) indicates a remarkable role of *sesn-1* in this process. RNA-interference experiments (Figure 6B and Table 2) further support *sesn-1*’s upstream role in the TORC1 pathway. Our studies (Figure 6C and Table 2) also suggest a possible interconnection between *sesn-1* and the *daf-16(FOXO)* transcription factor, independent of the insulin/*IGF* pathway. However, these studies should be interpreted with caution and require further evaluation.

Many of Sestrin’s functions appear to be mediated through its interactions with GATOR2. Our study showed that GATOR2-deficient worms failed to exhibit *sesn-1*-driven lifespan extension during CD, indicating GATOR2’s essential role in this pathway (Figure 4A, 4B and Table 1). Thus, Sestrins may primarily exert their lifespan-regulating and stress-protective effects through modulation of mTORC1 activity and autophagy. This proposition aligns with the studies in *D. melanogaster*, underscoring the interplay between BCAAs deprivation, *dSesn*, mTORC1 and autophagy [37]. BCAAs restriction has also been noted to improve metabolic health in humans [58].

Future endeavours targeting *sesn-1* deactivation in diverse cell types could shed light on its nuanced roles in regulating longevity and stress response. Elucidating the beneficial role of *sesn-1* in nematode lifespan extension has important implications for developing strategies to enhance lifespan and healthspan in humans, given the evolutionary conservation of Sestrins across metazoan species. Targeting Sestrins could pave the way for therapeutic interventions that mimic CR benefits, offering promising strategies for mitigating age-associated diseases and delaying aging.

## MATERIALS AND METHODS

### Strains of *C. elegans* and their maintenance conditions

Strains sourced from the Caenorhabditis Genetics Center (CGC) included: *C. elegans* Bristol N2 WT strain, RB2325 *sesn-1(ok3157)I* with deletion of 535bp in exon 3 of the *sesn-1* gene, DA2126 (*adls2122* (*lgg-1p*::GFP::LGG-1 + *rol-6*(*su1006*)), PD4251(*ccIs4251)I, e1282 IV* (*myo-3p*::GFP::LacZ::NLS + *myo-3p*::mitochondrial GFP + *dpy-20*(+)), CB1370 *daf-2*(*e1370*)*III*, DA1116 *eat-2*(*ad1116*)*II*, CF1038 *daf-16*(*mu86*)*I*, *E. coli* OP50 and HT115(DE3) strains. Additionally, IE24589 *sesn-1(ie24589)*, with an MOS-1 transposon insertion in exon 3 of the *sesn-1* gene, was kindly provided by Yohann Duverger, Universite Lyon. All strains underwent 8 outcrossing against the Bristol N2 WT strain to mitigate off-target mutations as outlined previously [59]. The *C. elegans* strains were maintained, synchronous culture was obtained and experiments were performed under standard conditions at 20°C [60].

### RNAi-expressing plasmid construction

RNA interference in *C. elegans* is a method to silence genes by introducing double-stranded RNA, which triggers degradation of a specific endogenous mRNA, effectively inactivating a target gene [61]. Total nematode RNA was extracted using Reagent ExtractRNA (Eurogen, Moscow, Russia) per manufacturer guidelines. Using reverse transcriptase Mint (Eurogen, Moscow, Russia), cDNA was synthesized and the subsequent cDNA fragments were PCR-amplified. The PCR primer pairs are shown in Table 3. The L4440 vector (Addgene, Watertown, MA, USA) was ligated with the respective cDNA fragments at XbaI and BamHI sites to create the RNAi-expressing constructs. Empty vector was used as control.

**Table 3:** Primer sequences for *C. elegans* candidate genes.

**Gene**	**Type**	**Sequence**
***sesn-1***	F	5′-agagagtctagaaccatgcacactac-3′
	R	5′-agagaggatcctcaatccaaagcctt-3′
***npp-18***	F	5′-agatctagagccagcgatatgacaatggcg-3′
	R	5′-attggatcctcgggcatggtagatcgaagac-3′
***Y32H12A.8***	F	5′-agatctagacgatctcatcgaaggtccatcg-3′
	R	5′-ataggatccccaccacctgtggcaataagc-3′

Abbreviations: F: Forward Prime; R: Reverse Primer.

### Bacteria-fed RNAi

Briefly, the transformed HT115 strain was grown in LB medium containing tetracycline (12.5 μg/ml) and ampicillin (100 μg/ml) at 37°C with shaking [62]. The bacteria were then plated on ampicillin-containing agarose plates supplemented with 1 mM IPTG and incubated at room temperature for two days. L1 *C. elegans* were added the following day. For double RNAi experiments, plates were prepared in a similar manner except that the 1:1 mixture of both RNAi bacterial clones was seeded into the plates.

### qPCR analysis of mRNA

Synchronized L1 nematodes were placed on bacteria-laden NGM plates. L3 worms were rinsed off the plates with M9, then cleansed three times with PBS. After washing the worms with PBS, they were suspended in the 0.1% Tween-20–PBS solution for 20 minutes to eliminate gut bacteria. The worms were snap-frozen in liquid nitrogen and RNA extraction was conducted using Reagent ExtractRNA (Eurogen, Moscow, Russia). A NanoDrop (Thermo Fisher Scientific Inc, Waltham, MA, USA) was used to determine RNA concentrations and the reverse transcription Mint Kit (Eurogen, Moscow, Russia) was utilized to synthesize cDNA from 3 μg of total RNA. mRNA levels were analyzed using the CFX96 (Bio-Rad Laboratories, Hercules, CA, USA) with qPCRmix-HS SYBR (Eurogen, Moscow, Russia) and assessed using the Bio-Rad CFX Manager software (Bio-Rad Laboratories, Hercules, CA, USA) (Supplementary Figure 3). Topoisomerase I (*top-1)* mRNA was used as a reference mRNA. The qPCR primer pairs listed in Table 4.

**Table 4:** Primer sequences for RNAi qPCR validation.

**Gene**	**Type**	**Sequence**
***sesn-1***	F	5′-tccgtgaagcaatttggaac-3′
	R	5′-tcgctaccatcattaccacg-3′
***npp-18***	F	5′-ttggcgcgttatttgggctc-3′
	R	5′-gttcttcggatccattgggattct-3′
***Y32H12A.8***	F	5′-ccacgacgtcgtcaaacgg-3′
	R	5′-gttgcatgccaatcgaggc-3′
***top-1***	F	5′-ggcccagaagaagtacgacagactg-3’
	R	5′-tcgatggcccaacggaatttc-3′

Abbreviations: F: Forward Prime; R: Reverse Primer.

### Lifespan analysis during CD

Synchronized L1 nematodes were plated on either RNAi plates or empty vector control plates. Upon reaching L4, worms were transferred to RNAi plates, empty vector control plates or plates lacking bactopeptone and bacteria. Viability was assessed daily according to the established protocols [39, 40]. Worms that disappeared or dried on the plate wall were excluded from the analysis.

### M9 media, paraquat and hydrogen peroxide survival analysis

To analyze viability in M9 media, L1 nematodes were introduced to 6 cm Petri plates with a thin M9 layer and kept in a 20°C incubator with gentle shaking. Daily aliquots were plated on NGM to count live nematodes. For paraquat and hydrogen peroxide treatments, L4 nematodes were added to 24-well plates containing thin layers of M9 media enriched with either 300 μM paraquat or 4 mM hydrogen peroxide. Viability was determined as previously described.

### Fluorescent microscopy analysis of autophagy and muscle degeneration

Autophagosomes in seam cells of the *adls2122* strain, expressing the LGG-1::GFP fusion protein, were quantified in both wild-type and *sesn-1*(RNAi) L3 animals using a Leica DMI4000B inverted microscope (Leica Microsystems, Wetzlar, Germany) at 640× magnification, as previously described [63, 64]. The number of myocyte nuclei labelled with GFP in *ccIs4251* strain, which expresses a myo-3 promoter driving a nuclear-targeted GFP-LacZ fusion and myo-3 promoter driving mitochondrially targeted GFP, was analyzed in the same manner for worms at the L4 stage and at the 5-day-old adult stage.

### Immunoblotting

Normalized nematode lysates containing 10 mg of protein per sample were subjected to 15% PAGE, as previously described [63–65]. The primary antibodies used for these studies were: anti-GFP (#11814460001, Roche Pharma, Penzberg, Germany), anti-phospho-ribosomal protein S6 (#sc-54279, Santa Cruz Biotechnology, Dallas, TX, USA) and anti-actin (#sc-47778, Santa Cruz Biotechnology, Dallas, TX, USA). The specificity of the anti-phospho-ribosomal protein S6 antibody was confirmed using a blocking peptide (#sc-54279 P, Santa Cruz Biotechnology, Dallas, TX, USA), adhering to the provided protocol. Blot images were acquired using the ChemiDoc Imaging System (Bio-Rad Laboratories, Hercules, CA, USA).

### Statistical analysis

Statistical analyses were performed using GraphPad Prism 10 software (GraphPad Software, Boston, MA, USA). One-way ANOVA with Bonferroni correction (*p* < 0.05) for multiple comparisons was used to assess the statistical significance of differences in mean lifespan across groups in all survival experiments. Lifespan tables show the *p*-value used to compare the studied groups with their controls. For autophagy and muscle degeneration analyses, Student’s unpaired two-tailed *t*-test was performed to compare the experimental groups with their respective controls. ns, ^*^, ^**^ and ^***^ correspond to *p*-values > 0.05, < 0.05, < 0.01 and < 0.001, respectively.

## Supplementary Materials

Supplementary Figures
