# Supplementary material for: Sesn-1 is required for lifespan extension during caloric deprivation in C. elegans through inhibition of mTORC1 and activation of autophagy
Source: Aging (Albany NY). 2025 Jul 28;17(7):1834–51. doi: 10.18632/aging.206290 (PMC12339033; doi:10.18632/aging.206290)
Supplement: Supplementary Figures [file aging-17-206290-s001.pdf]

SUPPLEMENTARY FIGURES

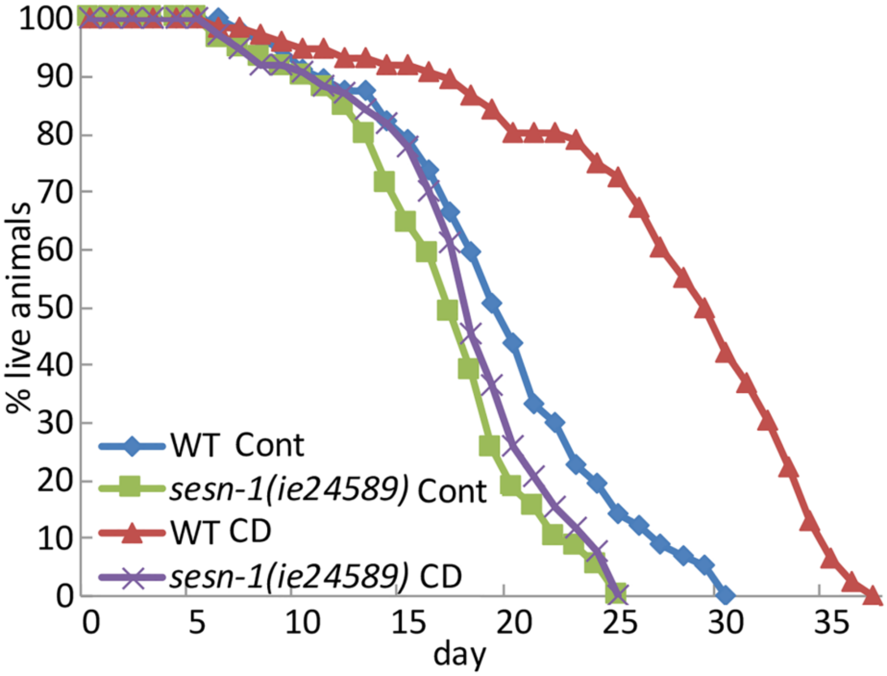

Supplementary Figure 1. *C. elegans* lifespan extension under CD is modulated by *sesn-1*. The lifespan of WT and *sesn-1(ie24589)* strain nematodes was assessed after plating animals on control (*n* = 57 WT, *n* = 57 *sesn-1(ie24589)* or axenic media (*n* = 76 WT, *n* = 77 *sesn-1(ie24589)*. The mean life expectancy of WT was 19.9 ± 0.74 days in control conditions and 27.9 ± 0.82 days in starvation conditions, $p >$ 0.001. The mean lifespan of *sesn-1(ie24589)* was 17,2 ± 0,59 days in control conditions and 18,1 ± 0,53 days in starvation conditions, $p < 0.05$.

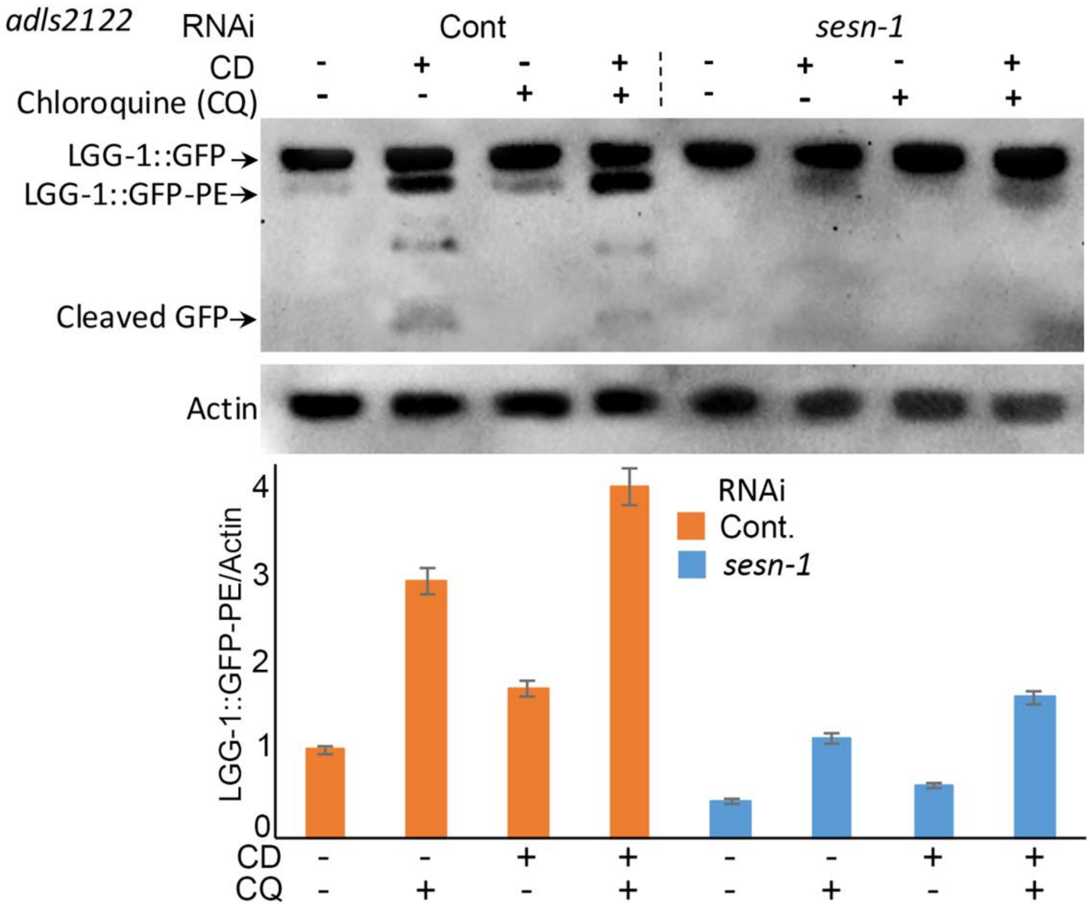

Supplementary Figure 2. Chloroquine treatment leads to increased autophagosome accumulation in *C. elegans* in a *sesn-1*-dependent manner. Control *adIs2122* and *adIs2122; sesn-1(RNAi)* nematodes were initially exposed to chloroquine and then shifted to CD conditions. The autophagosomal fraction of GFP::LGG-1 was assessed using immunoblot densitometry. All bar graphs represent blot intensity normalized to actin.

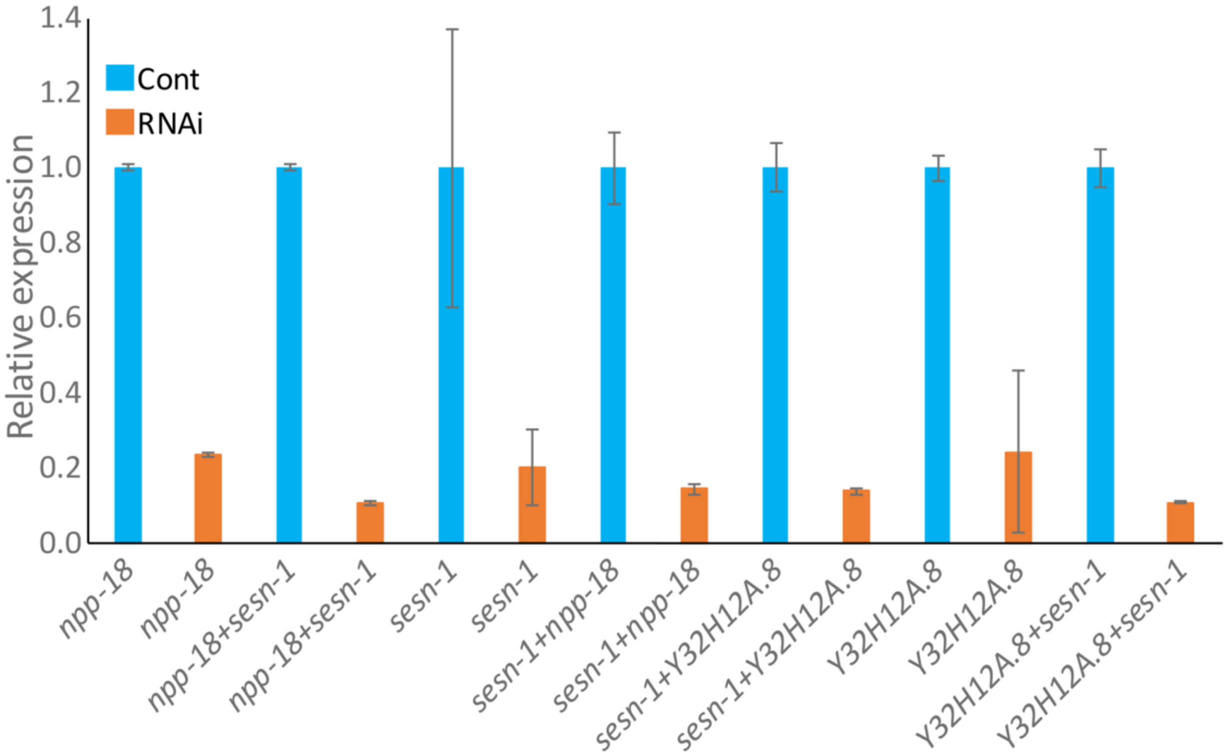

Supplementary Figure 3. qPCR validation of RNA interference targeting *sesn-1*, *npp-18*, and *Y32H12A.8* in WT and *sesn-1(ok3157)* nematodes. All bar graphs represent relative gene expression normalized to the *top-1* mRNA.
